# Exceptionally Efficient and Recyclable Heterogeneous Metal–Organic Framework Catalyst for Glucose Isomerization in Water

**DOI:** 10.1002/cctc.201701825

**Published:** 2018-01-08

**Authors:** Ryan Oozeerally, David L. Burnett, Thomas W. Chamberlain, Richard I. Walton, Volkan Degirmenci

**Affiliations:** ^1^ School of Engineering University of Warwick Coventry CV4 7AL UK; ^2^ Department of Chemistry University of Warwick Coventry CV4 7AL UK

**Keywords:** biomass, carbohydrates, heterogeneous catalysis, metal–organic frameworks, platform chemicals

## Abstract

Heterogeneous catalysts are desired for the conversion of glucose, the most abundant sugar in renewable biomass, but presently their synthesis requires highly toxic chemicals with long synthesis times. We report the conversion of glucose into fructose and 5‐hydroxymethylfurfural on a heterogeneous catalyst that is stable and selective and operates in the most environmentally benign solvent, water. We used a bifunctional solid with Lewis and Brønsted acid sites by partially replacing the organic linker of the zirconium organic framework UiO‐66 with 2‐monosulfo‐benzene‐1,4‐dicarboxylate. This catalyst showed high product selectivity (90 %) to 5‐hydroxymethylfurfural and fructose at 140 °C in water after a reaction time of 3 h. It was recyclable and showed only a minor loss in activity after the third recycle, offering a realistic solution for the bottleneck glucose isomerization reaction for scale‐up and industrial application of biomass utilization.

Sustainable production of chemicals requires the utilization of renewable resources, one of the most promising of which is lignocellulosic biomass.^1^, ^2^ Biomass‐derived sugars (e.g., glucose or fructose) can be converted into platform molecules, for example, 5‐hydroxymethylfurfural (HMF), which can be further processed into monomers, fuel additives, paints, and a variety of fine chemicals envisaged in a future biorefinery.^3^, ^4^ Although fructose can be converted into HMF easily,^5^ glucose is the main building block of lignocellulosic biomass, and its conversion remains challenging.^4^ The best‐performing heterogeneous catalyst for this conversion is tin‐incorporated beta zeolite (Sn‐beta) with Sn^4+^ occupying a fraction of the tetrahedral sites in the zeolite framework.^6^, ^7^, ^8^ Sn‐beta can effect the isomerization of glucose to fructose in water with high selectivity (>50 %).^7^ However, Sn‐beta requires long crystallization times, up to 40 days, which is industrially unviable at high temperatures, 140 °C, and, moreover, requires the use of hydrofluoric acid, an acute poison and extremely corrosive.^7^ In this work, we present a recyclable catalyst for glucose isomerization. It is based on modified UiO‐66 (Figure 1 a),^9^ a thermally and hydrothermally robust metal–organic framework (MOF), which we show matches the conversion and product selectivity of Sn‐beta.

**Figure 1:**
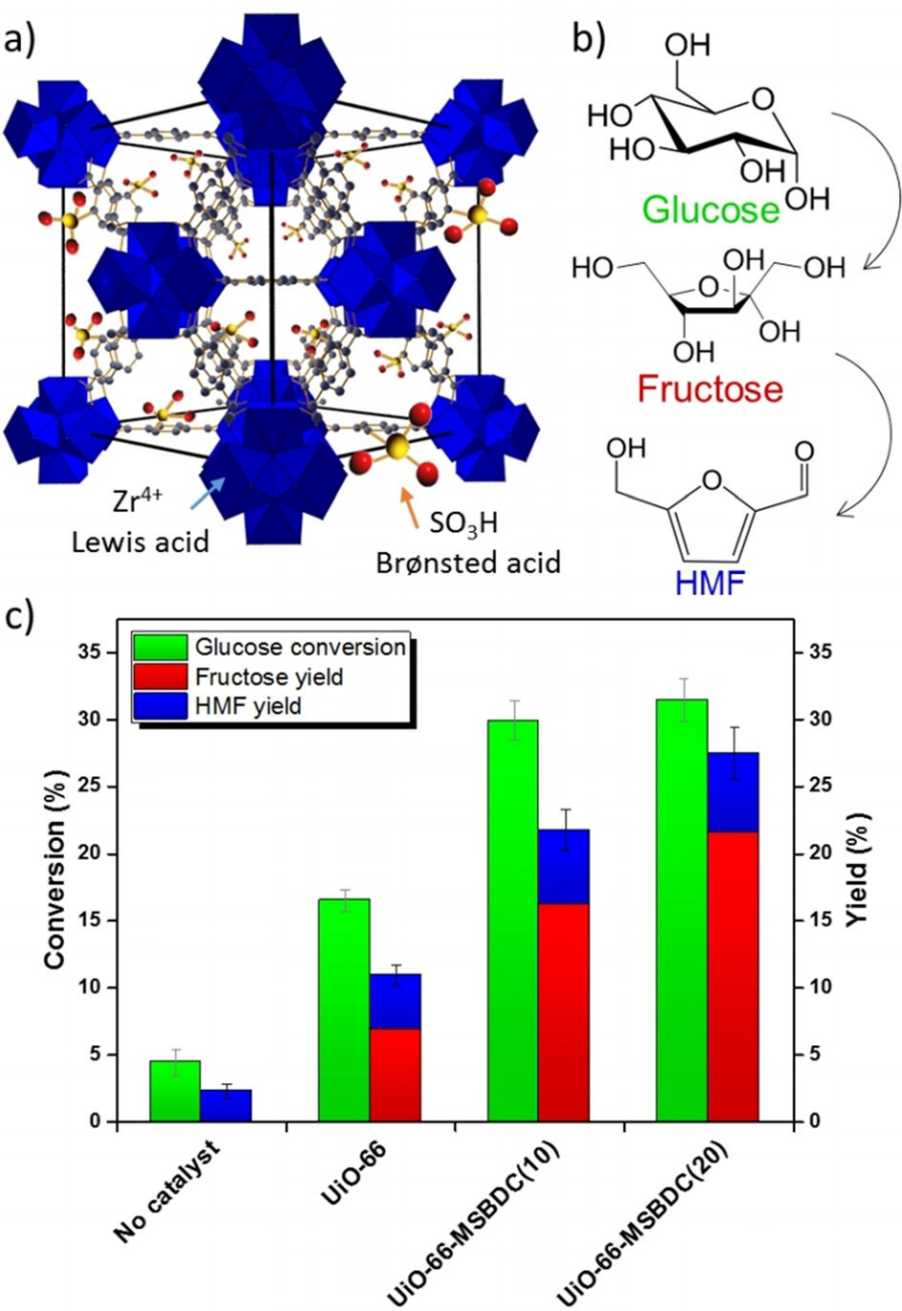
a) Schematic representation of the UiO‐66 framework. b) Glucose conversion into HMF through isomerization into fructose. c) Isomerization of glucose in water on metal–organic framework catalysts: UiO‐66, UiO‐66‐MSBDC(10) and UiO‐66‐MSBDC(20). Reaction conditions: 140 °C, 3 h, stock solution of 10 wt % glucose in deionized water.

The advantage of using MOFs as heterogeneous catalysts is the potential for tuning the solids’ properties by inclusion of desired functional ligands,^10^ such as acid sites,^11^ and at the same time by simple synthesis protocols, in this case without highly toxic and corrosive HF,^12^ in less than 24 h at 120 °C.

The challenge in HMF production from glucose is to achieve high product selectivity. The reaction proceeds through isomerization of glucose to fructose (Figure 1 b),^13^ which is the limiting step to achieve high selectivity. It was previously proposed that the reaction is catalyzed by Lewis acids,^13^ which enable hydride shift between the carbon atoms of glucose,^14^ and at the same time, proximal silanol groups or Brønsted acid sites form a hydrogen‐bonding network, which facilitates proton mobility.^15^ UiO‐66 is a zirconium‐based MOF with benzene‐1,4‐dicarboxylate (BDC) linkers, and it is highly stable in air up to 500 °C as is hydrothermally inert.^9^ Defects in the form of coordinatively unsaturated Zr^4+^ sites provide Lewis acidity.^16^ We found that UiO‐66 itself was active in glucose conversion (Figure 1 c) and showed 16 % conversion accompanied with 10 % product yield at 140 °C in 3 h. However, it lacks Brønsted acid sites. Therefore, we synthesized a catalyst by partially replacing the BDC linker with 2‐monosulfonated benzene‐1,4‐dicarboxylic acid (MSBDC),^17^, ^18^ and this catalyst showed 31 % glucose conversion under the same reaction conditions with 28 % product yield (Figure 1 c). This corresponds to exceptional product selectivity of approximately 90 %, which is similar to that previously reported for Sn‐beta zeolite.^7^

The ratio between the BDC and MSBDC linkers is critical for the successful synthesis of a stable, functionalized UiO‐66 material. Higher ratios of MSBDC within the framework were already shown to decrease the stability UiO‐66.^9^, ^18^ Indeed, we found that if only MSBDC was used as the ligand then the material subsequently collapsed upon hydrothermal treatment (Figure S1 in the Supporting Information). As such, materials containing 10 and 20 % functionalized linker were synthesized [UiO‐66‐MSBDC(*y*), in which *y* represents the mol % of MSBDC linker of the total linker content]. Scanning electron microscopy (SEM) images (Figure 2 a, c) show the particle morphology of UiO‐66 and UiO‐66‐MSBDC(20). Zirconium energy‐dispersive X‐ray (EDX) mapping (Figure 2 b, d) demonstrates the uniform distribution of zirconium atoms in both MOF structures, whereas sulfur EDX mapping of the UiO‐66‐BDC(20) catalyst (Figure 2 e) indicates a similar distribution of the modified linker across the MOF crystal. Although EDX mapping analysis does not give information on the three‐dimensional distribution, it clearly implies the uniform distribution of Brønsted acid sites with some evidence for enrichment at the crystal surface of the UiO‐66‐MSBDC(20) catalyst (Figure S2 for all catalysts). Further, EDX analysis of the MSBDC‐containing materials revealed the absence of sodium, supported by bulk inductively coupled plasma optical emission spectrometry (ICP‐OES) analysis, consistent with the displacement of sodium ions during synthesis to yield Brønsted acidic SO_3_H sites.

**Figure 2:**
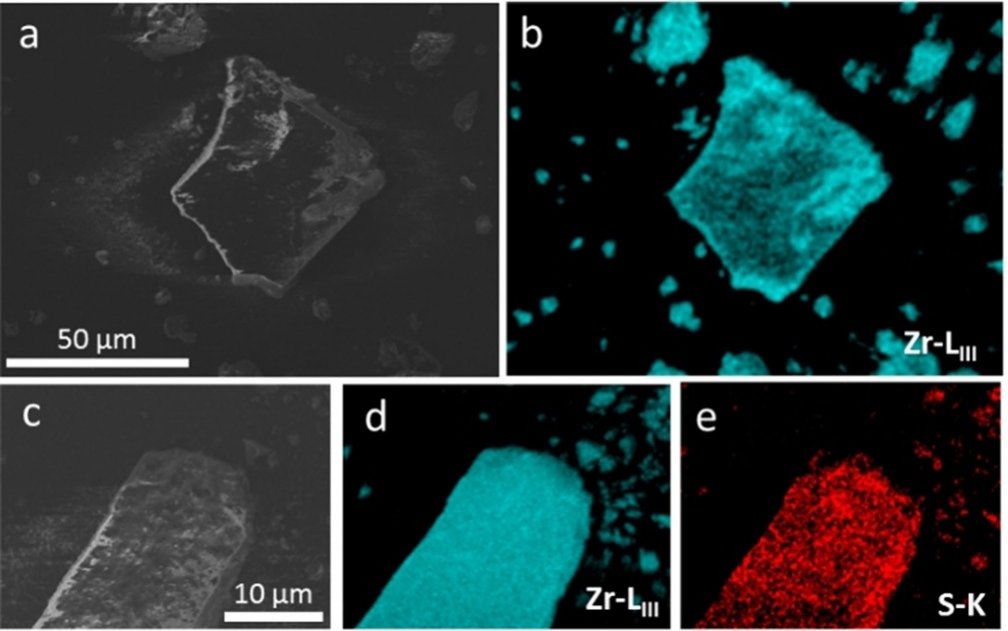
a) SEM image and b) zirconium EDX mapping of UiO‐66. c) SEM image and d) zirconium and e) sulfur EDX mappings of UiO‐66‐MSBDC(20).

The incorporation of sulfonic acid groups was also confirmed through FTIR spectroscopy. New signals at $\tilde{\nu }$ =620, 1078, 1180, and 1223 cm^−1^ appear in the spectra of the UiO‐66‐MSBDC catalysts, and their intensities increase upon increasing the linker content (Figure S3). These bands are attributed to the characteristic asymmetric bending and symmetric and asymmetric stretching vibrations of the S=O and S−O bonds.^19^, ^20^ Elemental analyses of the fresh catalysts also show S/Zr ratios close to the expected values (Tables S1 and S2). Thermogravimetric analysis (TGA) shows extensive mass loss at approximately *T*=510 °C for both the standard and functionalized UiO‐66 materials (Figure S4). This is consistent with the reported decomposition temperature of 540 °C for UiO‐66 and approximately 500 °C for sulfonic UiO‐66 materials reported.^9^, ^18^ The mass losses correlate to MSBDC linker contents of 14.6 and 24.7 % for UiO‐66‐MSBDC(10) and UiO‐66‐MSBDC(20) (Figure S4), respectively, which are close to the expected values. As a result, the ratios of zirconium/linker in UiO‐66, UiO‐66‐MSBDC(10), and UiO‐66‐MSBDC(20) are 5.51, 5.11, and 5.63, respectively, and thus, coordinatively unsaturated Zr^4+^ sites are present (Tables S3 and S4).

Powder X‐ray diffraction (PXRD) analysis of the catalysts shows the formation of crystalline MOF structures (Figure 3 a). Indeed, the addition of MSBDC does not alter the average structure of UiO‐66. The lattice parameter of fresh UiO‐66 is determined as 20.7516(2) Å (Figure S5). This value compares well with the reported value of 20.7551(5) Å,^9^ whereas the lattice parameter of UiO‐66‐MSBDC(20) is determined as 20.7431(13) Å (Figure 3 a), and a similar result is obtained for UiO‐66‐MSBDC(10) (Figure S5).

**Figure 3:**
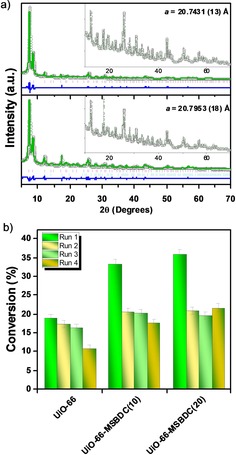
a) PXRD patterns of fresh UiO‐66‐MSBDC(20) (top) and after the fourth run (bottom). Insets show the 2 *θ* region between 10 and 70°. The green lines are the fitted profile, the black dots are the observed data, and the blue line is the difference in the two patterns. The ticks represent positions of allowed Bragg peaks: pink for UiO‐66 and pale blue for 1,4‐benzenedicarboxylic acid. b) Glucose conversion after the recycling tests.

Significant increases in the fructose yields combined with marginal increases in the HMF yields suggest that modification of UiO‐66 with MSBDC could affect the Lewis acidity in two ways. First, more defective materials are formed; this is supported by an increase in the mesopore volume of the UiO‐66‐MSBDC catalysts (Table S5 and Figure S6). Second, the Lewis acidity of Zr^4+^ is known to be enhanced significantly by the presence of a nearby electron‐withdrawing group; this was previously extensively studied in sulfated zirconia catalysts.^21^ This effect was recently reported in MOFs in the presence of electron‐withdrawing functional groups such as NO_2_ on the organic linker;^22^ indeed, we found that the conversion over NO_2_‐modified UiO‐66 was higher than that over the parent material (Figure S8), and so it is conceivable that the sulfonyl acid groups have a similar effect. Clearly, additional work is needed to understand fully the interplay of the acid functionalities.

The recyclability of catalysts is crucial for scale‐up and industrial applications: we studied this by recovering the solid catalysts by using a centrifuge and washing them with water after each reaction cycle. Full recovery of the catalysts was not possible owing to the presence of small catalyst particles that remained dispersed in the reaction medium. These nanocrystalline catalysts have intrinsically high activity (Figure S8). However, once the small particles were filtered out after the first run, the catalysts could be recovered in consecutive reaction cycles (Table S6). Therefore, although a decrease in glucose conversion was observed after the first run, no loss in activity was observed in the following three recycles (Figure 3 b), particularly for the UiO‐66‐MSBDC(20) catalyst (see Table S9 for product yields). The PXRD pattern of UiO‐66‐MSBDC(20) recovered after four runs showed that the integrity of the MOF lattice was maintained (Figure 3 a). Zirconium and sulfur EDX mapping of the catalysts after four reaction cycles further confirmed the integrity of the recycled catalysts (Figure S2). Recycling of the UiO‐66 and UiO‐66‐MSBDC(10) catalysts resulted in a minor loss of activity after the fourth run. This loss in activity could in part be a result of the formation of undesired side products, such as humins. These are poorly characterized oligomeric species that are known to be the main side products of this reaction.^3^ These insoluble products can accumulate on the catalyst surface and block the active sites. Indeed, the recovered catalyst mass in the recycling test increased owing to the collection of inseparable side products (Table S3), which could explain why the recycled catalyst had a lower sulfur count than the fresh catalyst, as determined by EDX analysis.

Notably, however, the elemental analysis of the reaction solution after the first reaction cycle (3 h reaction at 140 °C) showed that only trace amounts of sulfur and zirconium were present, which confirmed the stability of the catalyst with negligible leaching during the reaction (Table S4). Finally, the performances of the UiO‐66 materials were compared to that of Sn‐beta. In the literature, Sn‐beta is used as a glucose isomerization catalyst with a Sn‐to‐glucose ratio of 1:50, and the catalyst weight of Sn‐beta far exceeds the amount of the MOF catalyst used in this study under similar reaction conditions, for which Sn‐beta showed 54 % glucose conversion with 30 % fructose yield.^7^ A similar conversion (48 %) and product yield (34 %, Figure S7) were obtained upon using 40 mg of the UiO‐66‐MSBDC(20) catalyst, an amount that was still less than a quarter of the amount of the Sn‐beta catalyst (200 mg).

Tailor‐made MOFs with desired functionalities have made it possible to achieve exceptionally efficient catalysts for glucose isomerization in water. UiO‐66‐MSBDC catalysts containing dual Lewis and Brønsted acidity provided exceptional product selectivity of approximately 90 % for the conversion of glucose into fructose and HMF, and this selectivity is close to that shown by Sn‐beta zeolite. Other MOF catalysts reported in the literature for glucose isomerization use frameworks constructed from toxic metals (e.g., chromium)^23^, ^24^, ^25^, ^26^ and/or have been used in non‐aqueous solvents that are toxic or flammable (e.g., DMSO or THF).^27^ Our results show that UiO‐66‐MSBDC(*y*) catalysts are highly promising for scale‐up because they operate under aqueous conditions and are recyclable, and furthermore, their synthesis is simple and short and does not require toxic and corrosive conditions. Scaled‐up synthesis of the MOFs by using continuous flow reactors, often using water as a reaction medium, makes this a realistic prospect.^28^ Enzymes including metal centers and basic histidine moieties possessing multifunctional capabilities are nature's catalysts, and they provide high selectivity at the expense of slow reactions and sensitive operational systems. Future work on MOF catalysts will be devoted towards better understanding of the active sites of these catalysts and the mechanism of their activity to optimize the product distribution and their long‐term stability under industrially relevant flow‐chemistry conditions.

## Experimental Section

### Synthesis of the catalysts

UiO‐66 was prepared by mixing zirconium chloride (2.481 g, Alfa Aesar), 1,4‐benzenedicarboxylic acid (3.54 g, Sigma–Aldrich), *N*,*N*‐dimethylformamide (100 mL, Fisher Scientific), and hydrochloric acid (37 %, 20 mL, VWR). The synthesis mixture was then transferred to a polytetrafluoroethylene‐lined (PTFE) autoclave and heated to 120 °C for 24 h. Afterwards, the material was filtered, washed with methanol, and dried in air at 70 °C. The UiO‐66‐MSBDC(*y*) catalysts were prepared by substituting benzene‐1,4‐dicarboxylic acid with monosodium 2‐sulfo‐benzene‐1,4‐dicarboxylate (TCI Chemicals).

### Catalytic activity tests

The catalyst (10 mg) was placed in a reaction vial (4 mL) with a magnetic stirring bar and 10 wt % aqueous glucose solution was added. The vial was closed and placed in a preheated oil bath at 140 °C for 3 h. The reaction was quenched at 0 °C, and the product mixture was analyzed by HPLC.

### Characterization of the catalysts

Powder XRD data were collected by using a PANalytical X'Pert Pro MPD equipped with monochromatic CuK_α1_ radiation and a PIXcel solid‐state detector. Micrographs and elemental maps were obtained by using a Zeiss Gemini scanning electron microscope with a large area SDD EDX detector operating at 5 keV. Nitrogen adsorption isotherms were measured at −196 °C with a Micromeritics ASAP2020 system. The samples were outgassed at 150 °C for 12 h prior to the sorption measurements. Infrared spectra were recorded by using a PerkinElmer Paragon 1000 FTIR spectrometer in attenuated total reflection mode. Thermogravimetric analysis (TGA) was performed by using a Mettler Toledo Systems TGA/DSC 1 instrument under a constant flow of air (50 mL min^−1^). Elemental analysis was performed by Medac Ltd (UK) for Zr and S by using ICP‐OES after digestion and for CHN by using combustion. Additional experimental details can be found in the Supporting Information.

## Conflict of interest

*The authors declare no conflict of interest*.

## Supporting information

As a service to our authors and readers, this journal provides supporting information supplied by the authors. Such materials are peer reviewed and may be re‐organized for online delivery, but are not copy‐edited or typeset. Technical support issues arising from supporting information (other than missing files) should be addressed to the authors.

Supplementary
