# Supplementary material for: Exceptionally Efficient and Recyclable Heterogeneous Metal–Organic Framework Catalyst for Glucose Isomerization in Water
Source: ChemCatChem. 2018 Jan 8;10(4):706–9. doi: 10.1002/cctc.201701825 (PMC5838531; doi:10.1002/cctc.201701825)
Supplement: Supplementary file 1 — Supplementary [file CCTC-10-706-s001.pdf]

Heterogeneous & Homogeneous & Bio- & Nano-
CHEMCATCHEM
CATALYSIS

Supporting Information

Exceptionally Efficient and Recyclable Heterogeneous Metal–Organic Framework Catalyst for Glucose Isomerization in Water

Ryan Oozeerally,[a] David L. Burnett,[b] Thomas W. Chamberlain,[b] Richard I. Walton,*[b] and Volkan Degirmenci*[a]

cctc_201701825_sm_miscellaneous_information.pdf

Contents:

A. Materials and Methods S2

A.1 Synthesis of materials S2

A.2 Catalytic activity tests S2

A.3 Recycle tests S2

A.4 Materials characterization S3

B. Supplementary Tables S4

C. Supplementary Figures S8

D. Supplementary Reference S14

A. Materials and Methods

A.1 Synthesis of materials

UiO-66 was prepared by adding $ZrCl_4$ (2.481 g, Alfa Aesar) and 1,4-benzenedicarboxylicacid (3.54 g, Sigma Aldrich) to a PTFE-lined autoclave with an internal volume of 150 ml. To this *N*,*N*-dimethylformamide (100 ml, Fisher Scientific) and then hydrochloric acid (37 %, 20 ml, VWR) were added. The reaction mixture was then stirred for 5 minutes to homogenise the mixture before been heated at 120 °C for 24 hours. Materials with sulfonic acid groupswere prepared in the same fashion but a portion of the benzene-1,4-dicarboxylicacid used in the synthesis was substituted with monosodium 2-sulfo-benzene-1,4-dicarboxylate acid (TCI Chemicals). The materials were then collected using a centrifuge, at which point the collected material was stirred in 200 ml of methanol (Fisher Scientific) for 48 hours to remove any DMF trapped in the pores of the MOF. The solid materials were then collected using a centrifuge, followed by decantation of the liquid, and then dried at 70 °C overnight to remove excessmethanol.

A.2 Catalytic activity tests

In a typical reaction 10 milligrams of heterogeneous catalyst was added to a 4 mL vial. A magnetic stirrer was added to the vial along with 3 mL of a stock solution of 10 wt. % glucose in deionized water. The vial was crimp sealed and placed in a preheated oil bath at 140 °C for 3 hours. After the reaction, the vial was removed from the oil bath and quenched in ice bath at 0 °C to stop the reaction. The reaction solution was then filtered using a hydrophobic syringe filter. Analysis of the filtered reaction solution was performed using a Shimadzu High Performance Liquid Chromatography unit (HPLC) fitted with a Bio-RAD HPX-87H column. Glucose and fructose were quantified using an ELSD detector while HMF was quantified using a PDA detector (note that no analysis for other sugar products was performed in the current work). The mobile phase used in the HPLC was an aqueous solution consisting of 5 mM sulfuric acid. The flow rate was set as 0.5 mL/min. The temperatures of the HPLC column oven, PDA cell, and ELSD detector were 65 °C, 40 °C and 65 °C, respectively.

A.3 Recycle tests

Recycle reactions were conducted in a 25mL reactor with PTFE lining (Berghof, BR-25). In a typical reaction, 50 mg of catalyst and a magnetic stirring bar was placed into the reactor. 15 mL of a solution of 10 wt. % glucose in deionized water was then added. The reactor was sealed and pressurized to 10 bar with helium. The reactor was brought to reaction temperature (140 °C) by placing it into a preheated aluminum block heated *via* an IKA heating/stirring plate. At the end of the reaction (3 hours), the reactor was removed from the heating block and quenched in an ice bath at 0°C to stop the reaction. The reactor was then depressurized and opened. The solid catalyst was recovered from the reaction solution using a centrifuge and washed with deionized water. The reaction solution was filtered and analyzed using a Shimadzu HPLC as described above (Section A2). In the following reaction tests, the recovered catalyst was added back into the 25mL reactor along with fresh stock solution. The

reaction procedure was then repeated under the same conditions in order the test the recyclability of the catalyst and products were analyzed as described above (Section A2).

A.4 Materials characterisation

Powder XRD data were collected using a Panalytical X'Pert Pro MPD equipped with monochromatic Cu Kα1 radiation and a PIXcel solidstate detector.

Nitrogen adsorption isotherms were measured at -196°C on a Micromeritics ASAP2020 system. The samples were outgassed at 150°C for 12 h prior to the sorption measurements. Brunauer-Emmett-Teller (BET) equation was used to calculate the specific surface area from the adsorption data obtained (P/P_0=0.05-0.25). The mesopore volume was calculated by Barrett-Joyner-Halenda (BJH) method on the adsorption branch of the isotherm. The micropore volume was calculated from the t-plot curve at thickness range between 3.5 and 5.4 Å.

Infra-red spectra were recorded using a Perkin Elmer Paragon 1000 FT-IR Spectrometer in attenuated total reflection mode.

Thermogravimetric analysis (TGA) was performed using a Mettler Toledo Systems TGA/DSC 1 instrument under a constant flow of air (50 mL/min). Differential scanning calorimetry (DSC) curves were also recorded. Data were recorded from room temperature up to 1000 °C at a rate of 10 °C/min.

Elemental analysis was performed by Medac Ltd (UK) for Zr and S using ICP-OES after digestion and for CHN using combustion.

Micrographs and elemental maps were obtained using a Zeiss Gemini scanning electron microscope with a large are SDD EDX detector, operating at 5 keV. AZtec analysis software was used to produce elemental maps, using data obtained from both the Zr LIII-edge and the S K-edge.

B. Supplementary Tables

Table S1. Elemental composition of fresh catalysts.

Catalyst	Carbon (wt. %)	Hydrogen (wt. %)	Nitrogen (wt. %)	Sulfur (wt. %)	Zirconium (wt. %)
UiO-66	30.77	3.20	<0.10	N/A	26.07
UiO-66-MSBDC(10)	30.02	3.04	0.81	1.19	26.85
UiO-66-MSBDC(20)	30.29	3.13	1.23	2.09	25.56

Table S2. Molar composition of fresh catalysts normalised with respect to zirconium content.

Catalyst	Carbon	Hydrogen	Nitrogen	Sulfur	Zirconium
UiO-66	9.0	11.1	<0.02	N/A	1.0
UiO-66-MSBDC(10)	8.5	10.3	0.19	0.13	1.0
UiO-66-MSBDC(20)	9.0	11.1	0.31	0.23	1.0

Table S3. Mass loss in the TGA measurements of catalysts and their corresponding molecular formula calculated from the mass loss. (In the molecular formula, n was determined as 5.51, 5.11 and 5.63 for the catalysts, UiO-66, UiO-66-MSBDC(10), and UiO-66-MSBDC(20) respectively).

Catalyst	Temperature (°C)	MassMeasured (%)	Molecular weight (g/mol)	Molecular formula
UiO-66	25	100.00	1946.3	$Zr_6O_4(OH)_4(BDC)_n(OH)_{2(6-n)}(MeOH)_x$
	370 °C	79.44	1546.1	$Zr_6O_6(BDC)_n(OH)_{2(6-n)}$
	1000 °C	37.98	739.2	$6{\cdot}ZrO_2$
				n = 5.51
UiO-66-MSBDC(10)	25 °C	100.00	1946.3	$Zr_6O_4(OH)_4(0.9{\cdot}BDC0.1{\cdot}MSBDC)_n(OH)_{2(6-n)}(MeOH)_x$
	370 °C	78.27	1523.3	$Zr_6O_6(0.9{\cdot}BDC0.1{\cdot}MSBDC)_n(OH)_{2(6-n)}$
	1000 °C	37.98	739.2	$6{\cdot}ZrO_2$
				n = 5.11
UiO-66-MSBDC(20)	25 °C	100.00	2122.3	$Zr_6O_4(OH)_4(0.8{\cdot}BDC0.2{\cdot}MSBDC)_n(OH)_{2(6-n)}(MeOH)_x$
	370 °C	78.09	1657.3	$Zr_6O_6(0.8{\cdot}BDC0.2{\cdot}MSBDC)_n(OH)_{2(6-n)}$
	1000 °C	34.83	739.2	$6{\cdot}ZrO_2$
				n = 5.63

Table S4. Theoretical formula of the catalysts. The total linker content, methanol (MeOH) and*N*,*N*-dimethylformamide (DMF) was obtained from TGA measurements combined with the elemental composition determined by ICP-OES.

Catalyst	Molecular formula
UiO-66	$Zr_6O_4(OH)_4(BDC)_{5.51}(OH)_{0.98}(MeOH)_{10}$
UiO-66-MSBDC(10)	$Zr_6O_4(OH)_4(0.86\cdot BDC0.14\cdot MSBDC)_{5.11}(OH)_{1.78}(DMF)_{0.19}(MeOH)_{10}$
UiO-66-MSBDC(20)	$Zr_6O_4(OH)_4(0.75\cdot BDC0.25\cdot MSBDC)_{5.63}(OH)_{0.74}(DMF)_{0.31}(MeOH)_9$

Table S5. BET surface area and pore volume of the catalysts.

Catalyst	BET Surface Area ($m^2\cdot g^{-1}$)	Micropore Volume ($cm^3\cdot g^{-1}$)	Mesopore Volume ($cm^3\cdot g^{-1}$)
UiO-66	737	0.26	0.04
UiO-66-MSBDC(10)	1061	0.33	0.11
UiO-66-MSBDC(20)	823	0.22	0.29

Table S6. Recovery of MOF catalysts after recycle reactions.

Catalyst	Initial catalyst (mg)	Recovered catalyst after 4th run(mg)
UiO-66	50.1	44.4
UiO-66-MSBDC(10)	51.2	75.1
UiO-66-MSBDC(20)	50.6	62.1

Table S7. Zirconium and sulfur content in the reaction solution after 3 h reaction at 140°C.

Catalyst	Sulfur (wt. %)	Zirconium (ppm)
UiO-66	N/A	0.2
UiO-66-MSBDC(10)	<0.10	0.4
UiO-66-MSBDC(20)	<0.10	0.4

Table S8. Comparison of the zirconium and sulfurcontent of the catalystsbefore and after the reaction at 140°C for 3 h.

Catalyst	Before the reaction			After the reaction		
	S (wt. %)	Zr (wt. %)	Molar Ratio (S/Zr)	S (wt. %)	Zr (wt. %)	Molar Ratio (S/Zr)
UiO-66-MSBDC(10)	0.39	13.47	0.13/1	0.08	1.00	0.08/1
UiO-66-MSBDC(20)	0.53	12.49	0.23/1	0.13	1.00	0.13/1

Table S9. Glucose Conversion and HMF Yield obtained after recycling the catalysts.

Catalyst	Reaction cycle	Glucose conversion (%)	Fructose yield (%)	HMF Yield (%)	HMF Selectivity (%)	Product Selectivity (%)
UiO-66	1	19.1	5.7	4.9	55.5	25.7
	2	17.3	2.7	2.3	28.9	13.3
	3	16.4	2.6	2.3	29.9	14.0
	4	10.8	2.8	2.4	48.1	22.2
UiO-66-MSBDC(10)	1	33.2	18.6	7.6	78.9	22.9
	2	20.6	6.9	3.4	50.0	16.5
	3	20.3	7.2	3.5	52.7	17.2
	4	17.5	5.3	2.9	46.9	16.6
UiO-66-MSBDC(20)	1	35.9	21.7	7.9	82.5	22.0
	2	20.9	7.9	3.3	53.6	15.8
	3	19.5	9.5	3.4	66.2	17.4
	4	21.7	6.5	2.8	42.9	12.9

C. Supplementary Figures

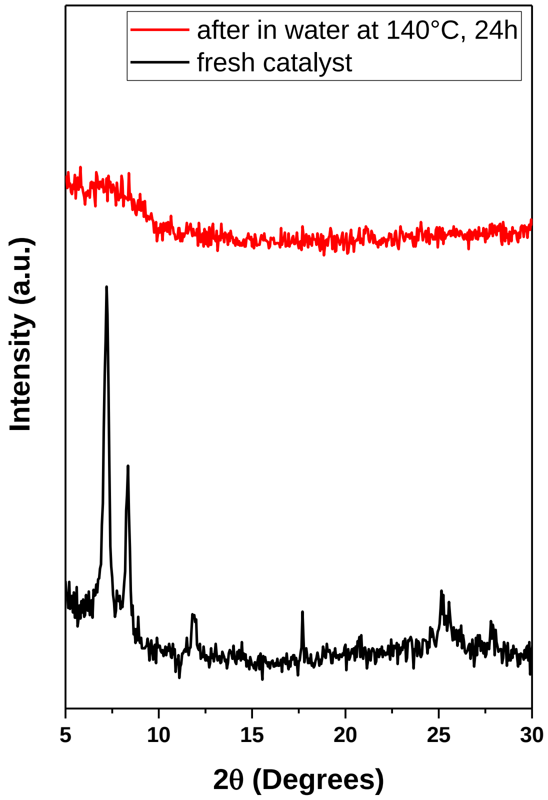

Figure S1. XRD powder diffraction pattern ofUiO-66-MSBDC(100); fresh catalyst (black) and after treatment in water, at 140°C for 24 h (red).

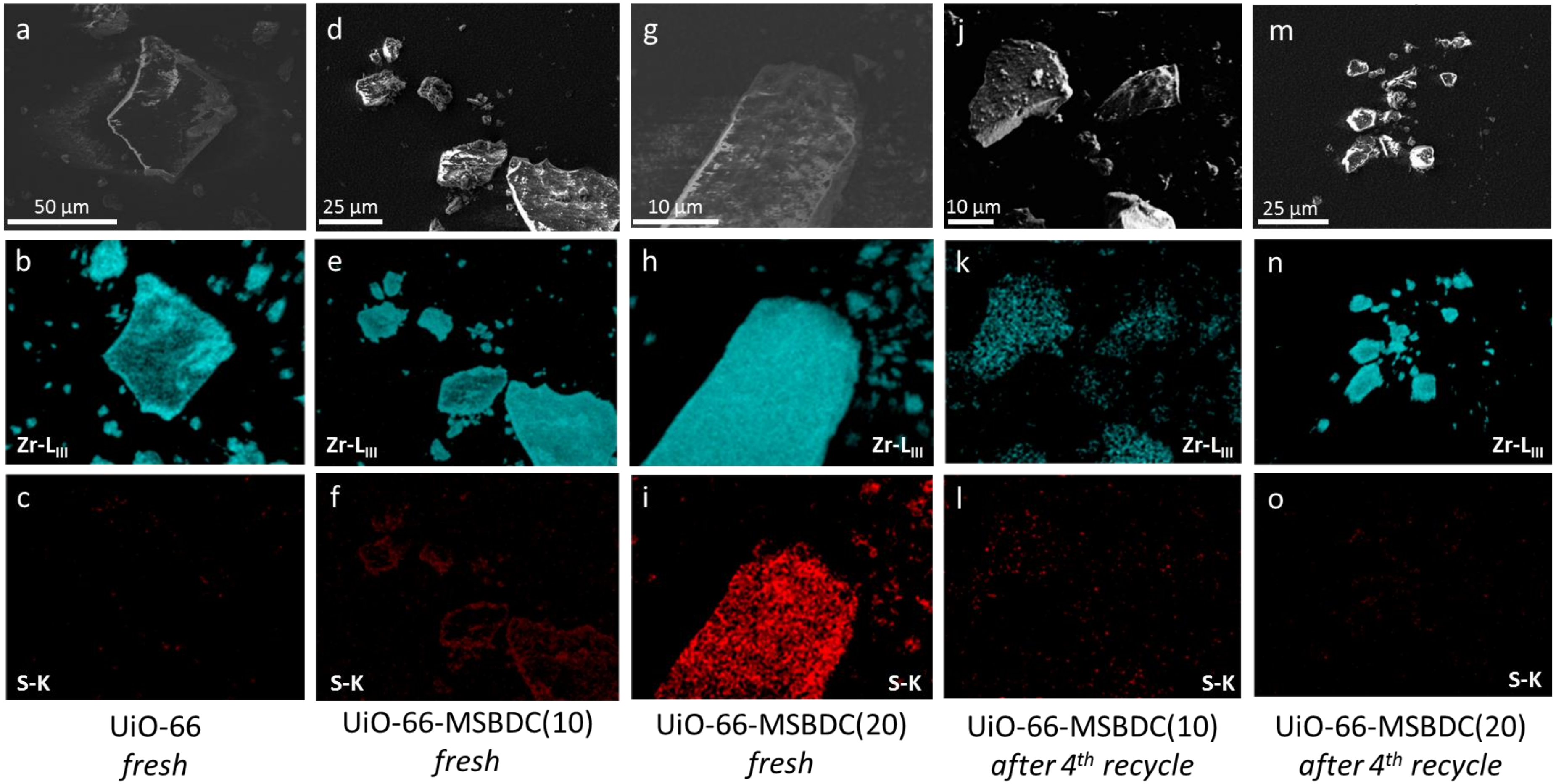

Figure S2. SEM Images(first row), EDX maps of zirconium (second row) and sulfur (third row) of fresh (a-i) and post-reaction catalysts (j-o).

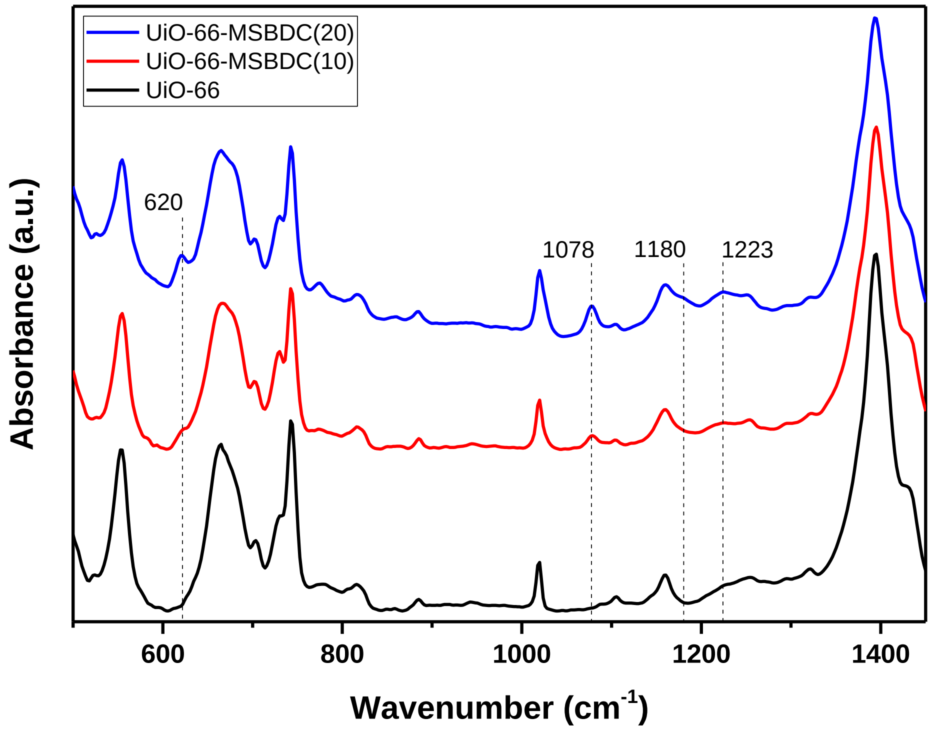

Figure S3. FT-IR spectra of the catalysts a) UiO-66 b) UiO-66-MSBDC(10) c) UiO-66-MSBDC(20). Characteristic asymmetric and symmetric stretching frequencies of S=O double bonds and S–O bonds are observed in the region of 900-1300 $cm^{-1}$and asymmetric bending at 620 cm^{-1}.

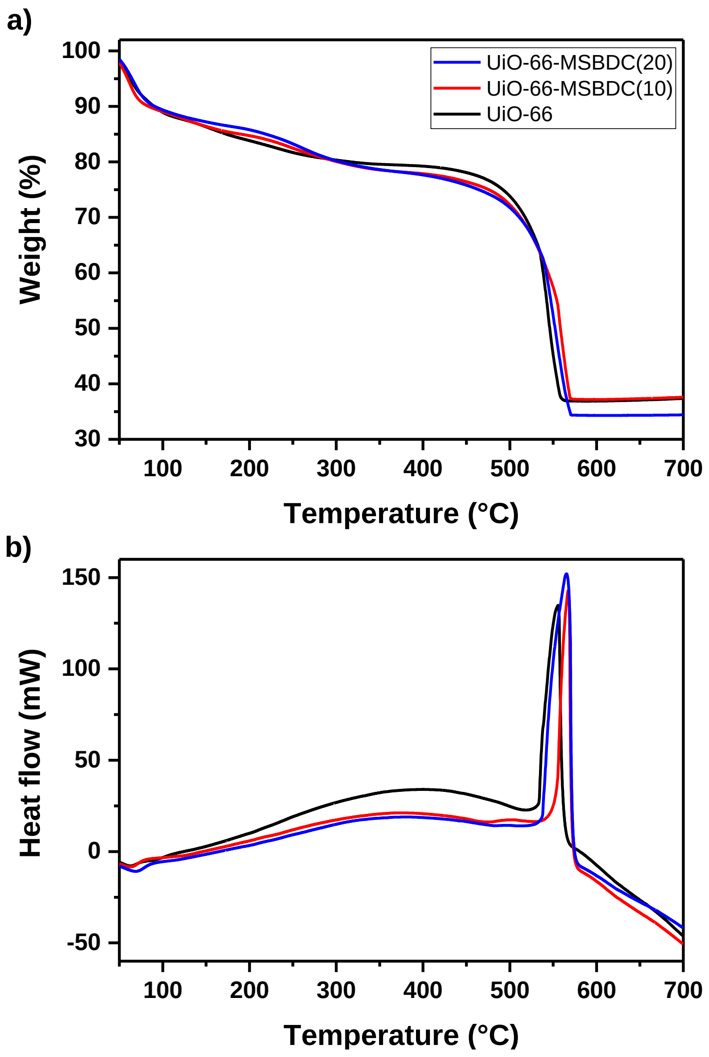

Figure S4. TGA (a) and DSC (b) curves of UiO-66, UiO-66-MSBDC(10) and UiO-66-MSBDC(20).

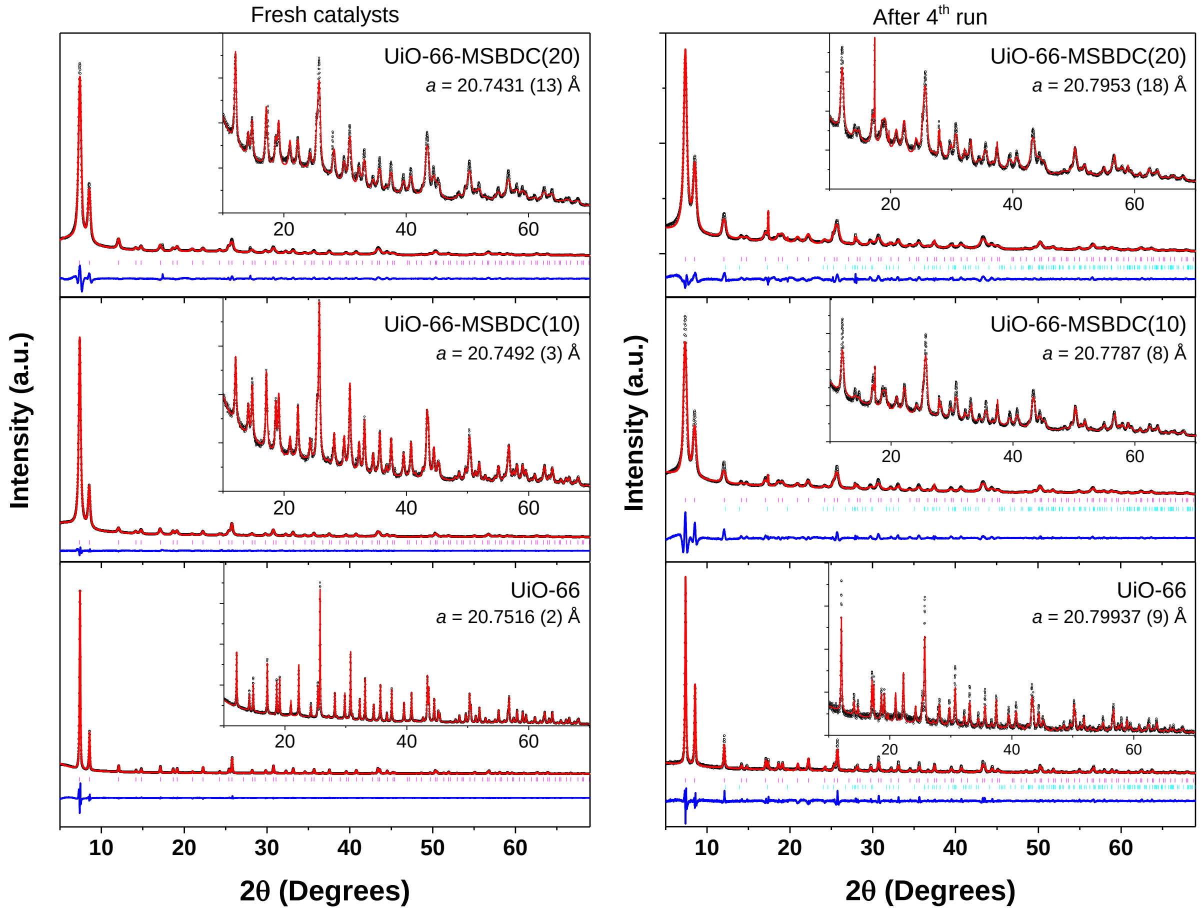

Figure S5. Powder XRD patterns of fresh catalysts (left) and after 4thrun (right); UiO-66, UiO-66-MSBDC(10), UiO-66-MSBDC(20). Insets show the 2 theta region between 10 and 70 degrees. The red lines are fitting, black dots are observed and blue line is the difference in two patterns.

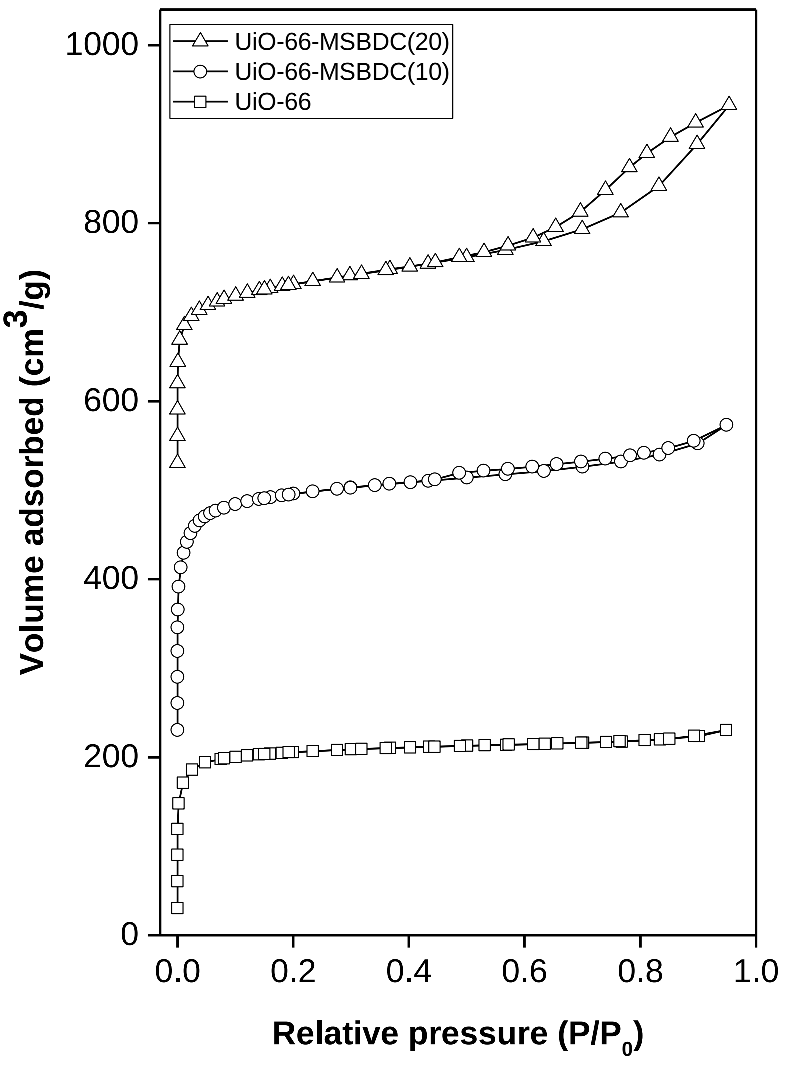

Figure S6. Nitrogen physisorption isotherms and BJH pore size distributions of the UiO-66, UiO-66-MSBDC(10) and UiO-66-MSBDC(20) catalysts.

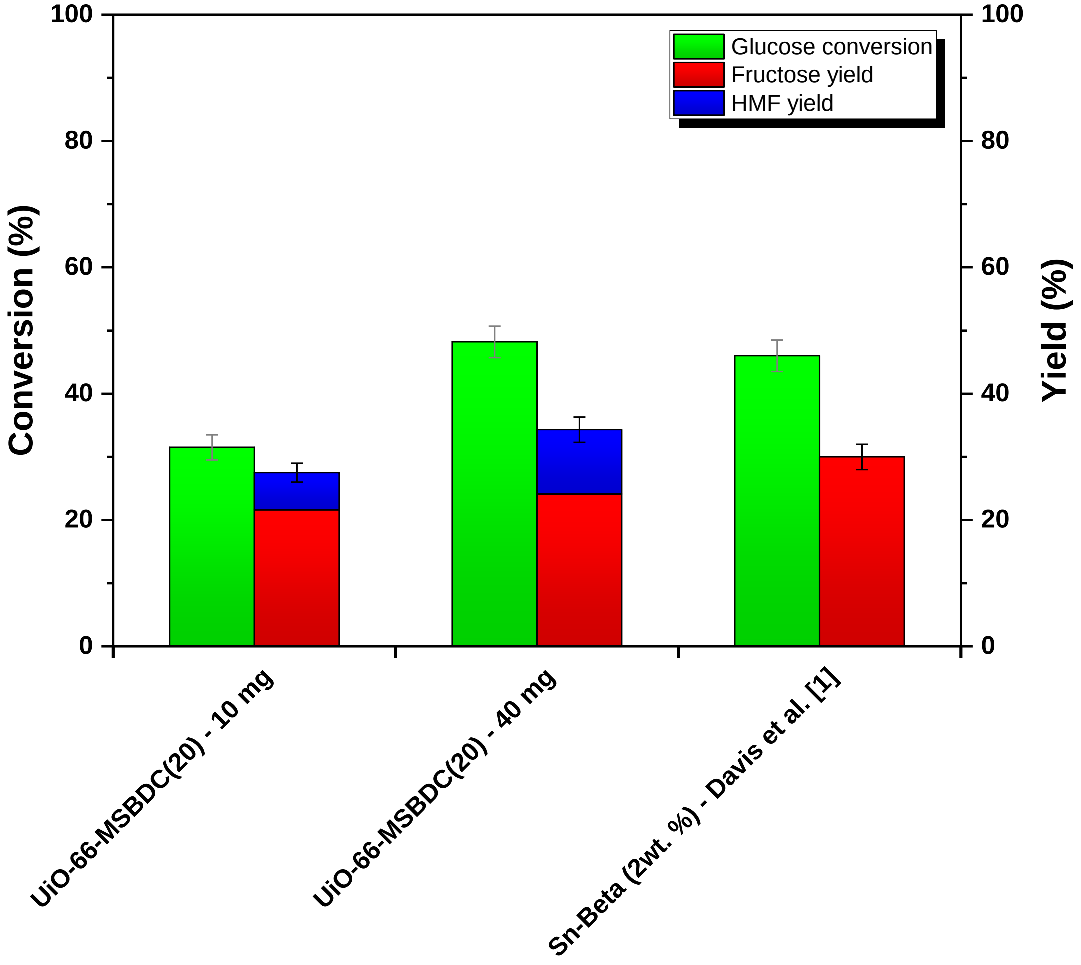

Figure S7. Glucose Conversion and HMF Yield on UiO-66-MSBDC(20) with 10 mg catalyst and fourfold increase of catalyst amount (40 mg) after 3h reaction and after 12 min of reaction. Sn-beta zeolite as reported in literature by Davis et al.[1] after 12 min reaction.

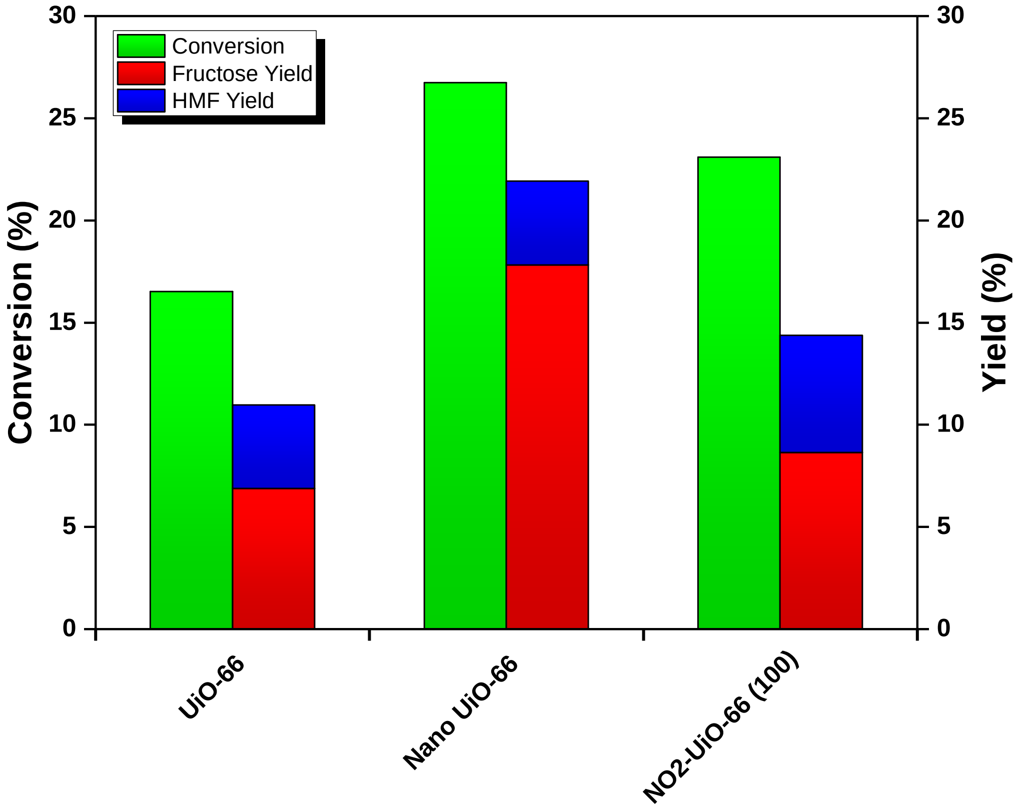

Figure S8. Glucose Conversion and HMF Yield on a) UiO-66-MSBDC b) Nano-crystalline UiO-66-MSBDC and c) UiO-66-MSBDC with NO2 functionality after 3 h reaction at 140 °C.

D. Supplementary Reference

1. Moliner, M.; Román-Leshkov, Y.; Davis, M. E., *Proc. Natl. Acad. Sci. U. S. A.* **2010,***107* (14), 6164-6168.
